# Robotics in neurointerventional surgery: a systematic review of the literature

**DOI:** 10.1136/neurintsurg-2021-018096

**Published:** 2021-11-19

**Authors:** William Crinnion, Ben Jackson, Avnish Sood, Jeremy Lynch, Christos Bergeles, Hongbin Liu, Kawal Rhode, Vitor Mendes Pereira, Thomas C Booth

**Affiliations:** 1 School of Biomedical Engineering and Imaging Sciences, King's College London, London, UK; 2 NIHR Biomedical Research Centre, Guy's and St Thomas' NHS Foundation Trust and King's College London, London, UK; 3 Department of Neuroradiology, King's College Hospital NHS Foundation Trust, London, UK; 4 Division of Neuroradiology, Department of Medical Imaging and Division of Neurosurgery, Department of Surgery, University Health Network - Toronto Western Hospital, Toronto, Ontario, Canada

**Keywords:** device, technology

## Abstract

**Background:**

Robotically performed neurointerventional surgery has the potential to reduce occupational hazards to staff, perform intervention with greater precision, and could be a viable solution for teleoperated neurointerventional procedures.

**Objective:**

To determine the indication, robotic systems used, efficacy, safety, and the degree of manual assistance required for robotically performed neurointervention.

**Methods:**

We conducted a systematic review of the literature up to, and including, articles published on April 12, 2021. Medline, PubMed, Embase, and Cochrane register databases were searched using medical subject heading terms to identify reports of robotically performed neurointervention, including diagnostic cerebral angiography and carotid artery intervention.

**Results:**

A total of 8 articles treating 81 patients were included. Only one case report used a robotic system for intracranial intervention, the remaining indications being cerebral angiography and carotid artery intervention. Only one study performed a comparison of robotic and manual procedures. Across all studies, the technical success rate was 96% and the clinical success rate was 100%. All cases required a degree of manual assistance. No studies had clearly defined patient selection criteria, reference standards, or index tests, preventing meaningful statistical analysis.

**Conclusions:**

Given the clinical success, it is plausible that robotically performed neurointerventional procedures will eventually benefit patients and reduce occupational hazards for staff; however, there is no high-level efficacy and safety evidence to support this assertion. Limitations of current robotic systems and the challenges that must be overcome to realize the potential for remote teleoperated neurointervention require further investigation.

## Introduction

Recent advances in engineering have led to the first robotically performed neurointerventional procedures, including diagnostic cerebral angiography, carotid artery intervention, and the treatment of intracranial aneurysms.^1^ There have been several narrative reviews on the topic,^1–3^ but no study has used an evidence-based approach to determine the safety and efficacy for robotically performed neurointervention. The aim of this systematic review therefore is to determine the range of indications and robotic systems together with the technical success, clinical success, and the degree of manual assistance required for robotically performed neurointervention. We will first discuss the potential benefits of using a robot for neurointervention. Robotic percutaneous coronary intervention (PCI) was first performed in 2005,^4^ and several of the systems used in neurointerventional studies were initially designed to perform cardiac and peripheral intervention. We therefore present a brief summary of previously available commercial devices for cardiovascular intervention which have either been adapted for neurointervention or represent a prototype on which a neurointerventional system could be developed. Definitions of some robotic terminology that clinicians may find useful are detailed in online supplemental table 1.

10.1136/neurintsurg-2021-018096.supp1 Supplementary data

### Why use a robot?

First, robotic surgery has the potential to allow the operator to perform procedures with greater precision, dexterity, and degrees of freedom while eliminating physiological tremors, operator fatigue, and allowing surgery to be performed in an optimum ergonomic position.^5^

Second, the use of robotic systems may reduce the occupational hazards to the operator, including radiation exposure and degenerative joint disease (particularly spinal), from wearing lead aprons.^6^ The ability to perform the procedure remotely may also reduce the potential transmission of infectious agents whether these are blood-borne pathogens from sharps injuries or airborne pathogens, including severe acute respiratory syndrome coronavirus 2.

Third, the use of teleoperated robots may allow the treatment of neurovascular disease from a remote location. This is of particular relevance to neurointerventional surgery, as a teleoperated platform could increase the number of eligible patients receiving mechanical thrombectomy (MT) for ischemic stroke. In the UK for example, 10% of all patients admitted for stroke are predicted to be eligible for MT,^7^ but currently only 1.4% of stroke admissions receive MT.^8^ This mismatch is primarily due to the relative paucity of trained operators and the widespread geographical distribution of ‘MT-capable’ stroke centers. MT is cost effective^9^ and level 1a evidence suggests that patients are more likely to achieve a good functional outcome after MT with a faster time to reperfusion.^10^ Recent data has also shown that patients arriving at a center unable to provide MT are likely to be deemed ineligible for MT if diagnosis and transfer is >3 hours.^11^ If proved to be safe, efficacious, and cost effective, a distributed network of teleoperated robotic systems could increase the number of eligible patients receiving MT who arrive at centers without a MT operator and additionally, improve the functional outcome for patients by reducing the delay to reperfusion.

### Endovascular robotic platforms applied to cardiac and peripheral vascular intervention

A summary of previous US Food and Drug Administration (FDA)-approved systems is shown in table 1, with their indication, mechanism of action, the advantages and potential limitations of each system. All current robotic platforms are controller-responder systems allowing control of interventional equipment by the operator from a workstation separated from the patient and protected from radiation.

**Table 1:** Summary of the previous commercial robotic systems used for cardiac and peripheral endovascular intervention, including the key advantages and disadvantages

Robotic system	Indication	Control panel	Method of catheter and guidewire manipulation	Advantages	Potential limitations
Sensei robotic system	Cardiac electrophysiological studies and treatment	Joystick	Steerable guide catheter inside a steerable sheath via tendon drives with movement in three dimensions	Steerable catheter allows precise movements to be performed more quickly than manually^51^	Requires the use of a bespoke sheath and requires the manual placement of EP catheter for recording or ablation
Niobe magnetic navigation system	Cardiac electrophysiological studies and treatment	Joystick/mouse	Fixed external magnets with a magnetic catheter tip and catheter advancer system	Magnetic control allows exceptional accuracy. Uses bespoke soft flexible catheter, reducing endovascular or cardiac injury^52^	Expensive, requiring large bespoke interventional theater to accommodate external magnets
Amigo remote catheter system	Cardiac electrophysiological studies and treatment	Handheld remote device	Three separate controllable mechanisms for linear motion, tip deflection, and rotation of catheter	No requirement for bespoke proprietary equipment in addition to robotic system	Specifically designed to manipulate EP catheters. limiting potential clinical translation to PCI or PVI
Magellan robotic system	Peripheral vascular intervention	Touchscreen, 3D joystick, foot pedal	Steerable guide catheter inside a steerable sheath via tendon drives with movement in three dimensions. Separate remote wire manipulator allowing linear and rotational movement of guidewire	System has been shown to increase procedure accuracy and reduce procedure time in vitro^53^ as well as reduce histological damage to vessels in vivo^54^	Requires the manual deployment of interventional devices and the use of bespoke proprietary catheter and sheath
CorPath 200	Coronary and peripheral vascular intervention	Touchscreen, joystick	Separate mechanisms for linear and rotational motion of guidewire. Mechanism for linear motion of rapid exchange catheter	Can use ‘off the shelf’ 0.014 inch coronary guidewires and rapid exchange catheters for intervention	No capability for guide catheter manipulation. Uses a disposable cassette that must be replaced between procedures

PCI, percutaneous coronary intervention; PVI, peripheral vascular intervention.

The approved robotic systems designed for cardiac electrophysiology illustrate several different ways a catheter can be robotically manipulated. The Sensei robotic system (Hansen Medical, Mountain View, USA) uses a proprietary tendon-driven guide catheter, which allows precise tip deflection followed by manual delivery of a cardiac mapping or ablation catheter. The Niobe magnetic navigation system (Stereotaxis, St Louis, USA) combines two fixed external magnets in the operating room, which set up a magnetic field across the patient. This is manipulated to control movements of a catheter containing a magnet at its tip. The Amigo remote catheter system (Catheter Robotics, Mount Olive, USA) uses ‘off the shelf’ electrophysiological catheters, unlike the Sensei or Niobe systems, and essentially reproduces the movements made by a cardiologist during a procedure. All systems have been shown to be safe and effective in small clinical trials.^12–14^

**Figure 1:**
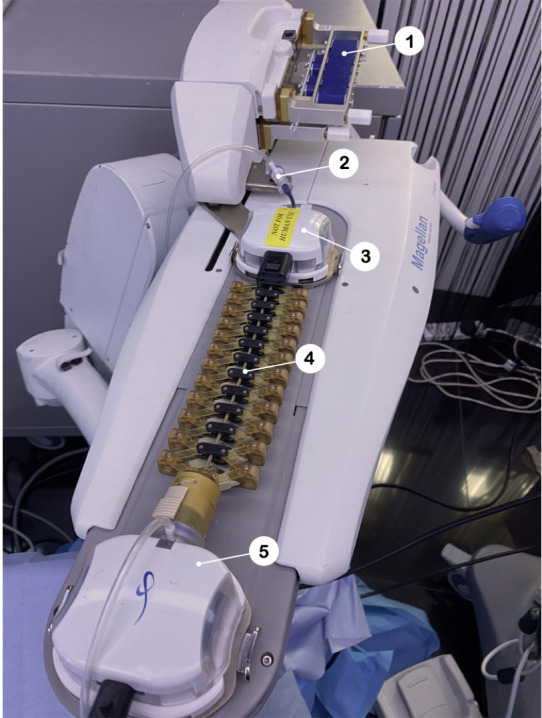
Robotic arm of the Magellan robotic system (Hansen Medical). This is one of two commercial systems to have been used for neurointervention. 1. Conveyor belt system for linear and rotational movement of guidewire. 2. Magellan steerable catheter. 3. Locking mechanism for steerable catheter. 4) Caterpillar-like control system for linear motion and steering of tendon driven catheter. 5. Locking mechanism for steerable sheath.

The Magellan robotic system (Hansen Medical) was the first commercially available robotic system to be used for peripheral vascular intervention (PVI) and has been used clinically in over 1000 cases, including carotid artery stenting (CAS), aortic aneurysm repair, peripheral arterial angioplasty, venous procedures, and a broad range of embolization procedures.^15^ The Magellan system can use either a 10 Fr, 9 Fr or 6 Fr Magellan robotic catheter to navigate to the target. The Magellan uses the same technology as the Sensei robotic system, that being tendons within the catheter to control deflection of the catheter tip. This adds an additional degree of freedom for the operator alongside linear and rotational movement. A feasibility study, with the system successfully treating 20 patients with symptomatic femoropopliteal disease,^16^ led to FDA approval of the Magellan system in 2012 for PVI. A diagram of the Magellan robotic arm is shown in figure 1.

**Figure 2:**
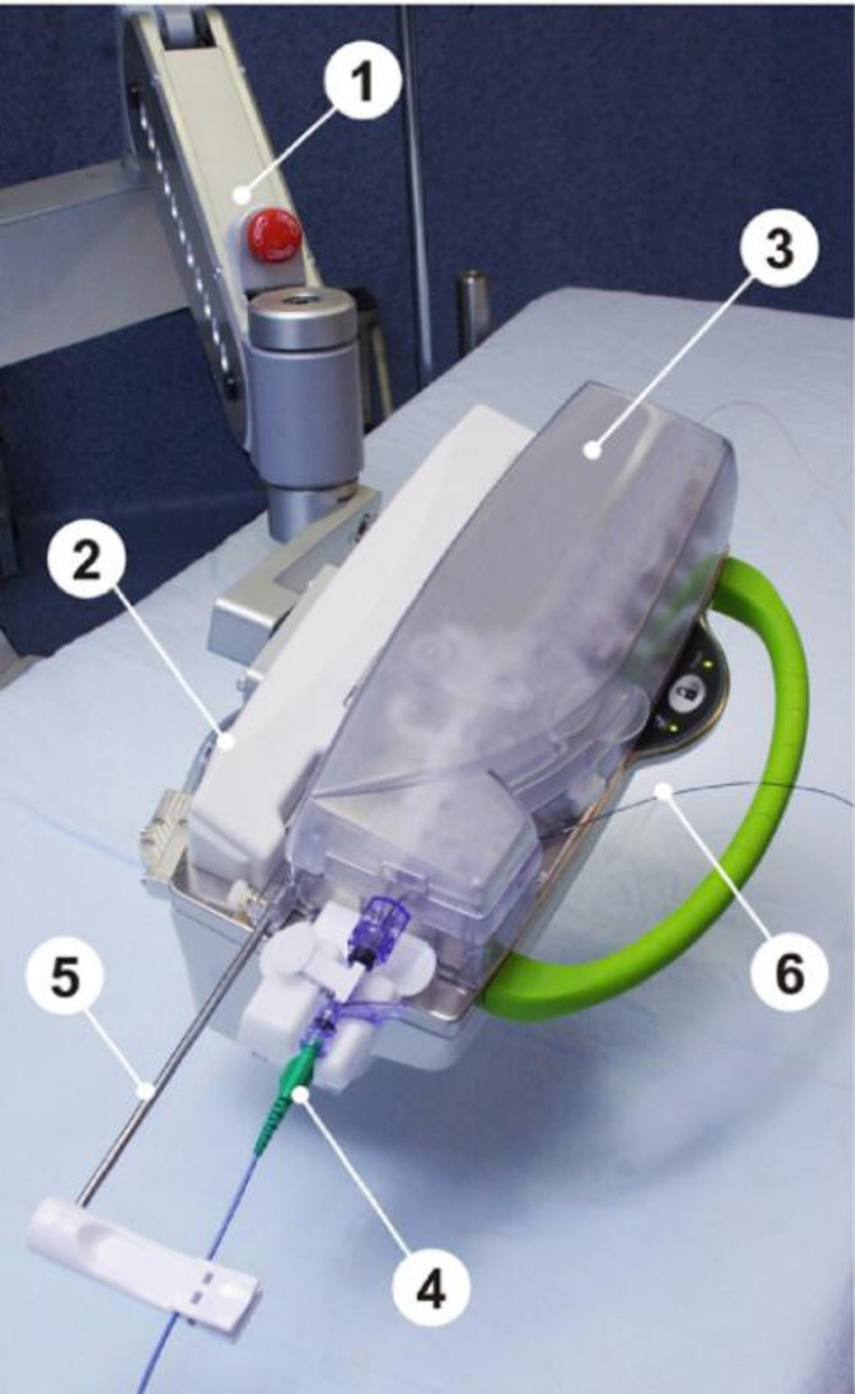
Robotic arm of the Corpath 200 robot (Corindus). 1. Articulated robotic arm. 2. Robotic drive. 3. Single-use sterile cassette. 4. Attachment of guide catheter. 5. Guide catheter support arm. 6. Loaded rapid exchange catheter. Reprinted with permission from Elsevier.^55^

**Figure 3:**
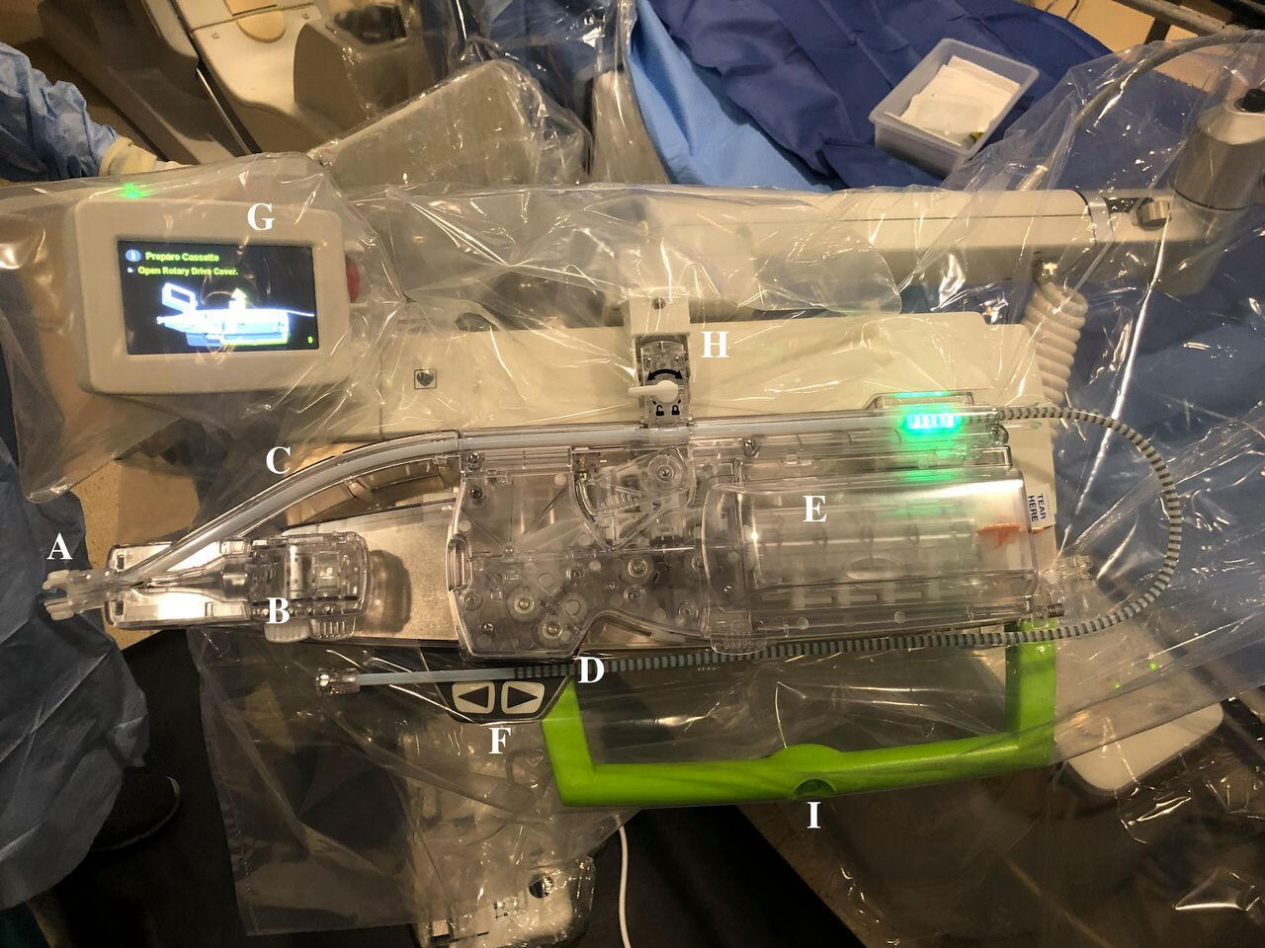
Corpath GRX robotic arm and cassette (Corindus). This is one of two commercial systems to have been used for neurointervention. A. Sheath attachment. B. Guide catheter rotation module. C. Guide support track. D. Rapid exchange device port. E. Guidewire rotation module. F. Micro-adjustment buttons. G. Robotic arm feedback console. H. Cassette locking mechanism. I. Robotic arm toggle button. Reproduced with permission from BMJ Publishing Group Ltd.^18^

The remote navigation system (Navicath, Israel) was the first robotic system which demonstrated feasibility for robotic PCI in humans.^4^ This device was the basis for the development of the Corpath 200 robot (Corindus, Waltham, USA), which subsequently received FDA approval for PCI and PVI. The robotic arm of the Corpath 200 is depicted in figure 2. One of the main limitations of the Corpath 200 system is the lack of active robotic control of the guide catheter, this being the main reason for the requirement of manual assistance during the robotic treatment of complex coronary lesions.^17^ To accommodate this, the next generation Corpath GRX (Corindus, Waltham, USA) system was modified by adding a separate mechanism allowing linear and rotational movement of the guide catheter. Both the Corpath 200 and Corpath GRX systems are compatible with 0.014 inch guidewires, rapid exchange balloons, and stent catheters. The system uses a disposable single-use sterile cassette, which leads to an increased cost of approximately US$300 per procedure.^18^ The layout of the Corpath GRX cassette is shown in figure 3.

While robotic systems have been approved for PCI and PVI, there has not yet been widespread use. A key issue is the lack of clinical evidence with currently no randomized control trial performed for any robotic catheter system to demonstrate a difference in clinical outcomes. Further problems which limit the uptake are the cost of systems together with the lack of well-designed economic studies and that while shielding the operator from radiation, the interventional staff are still required to stay in the radiation field to change interventional equipment within the robotic arm and manually deploy interventional devices.^19^ Corindus currently manufacture the Corpath GRX system and are running clinical trials for cardiac and neurointervention; however, the Magellan robotic system has been discontinued after Hansen Medical was acquired by Auris Health. This is potentially due to current commercial focus as Auris Health manufacture the Monarch system, which is intended for use in robotic bronchoscopy.

## Methods

The Preferred Reporting Items for Systematic Reviews and Meta-Analyses (PRISMA) guidelines were followed for the reporting of this review.^20^ We searched Medline, PubMed, Embase, and Cochrane databases to identify peer-reviewed articles reporting robotically performed neurointervention. Our eligibility criteria were any article which reported the outcomes following robotically performed neurointervention for intracranial procedures, diagnostic cerebral angiography, and CAS. The medical subject headings (MeSH) terms used to perform the literature search are provided in the online supplemental appendix. All searches were performed on articles published up to and including April 2021.

10.1136/neurintsurg-2021-018096.supp2 Supplementary data

**Figure 4:**
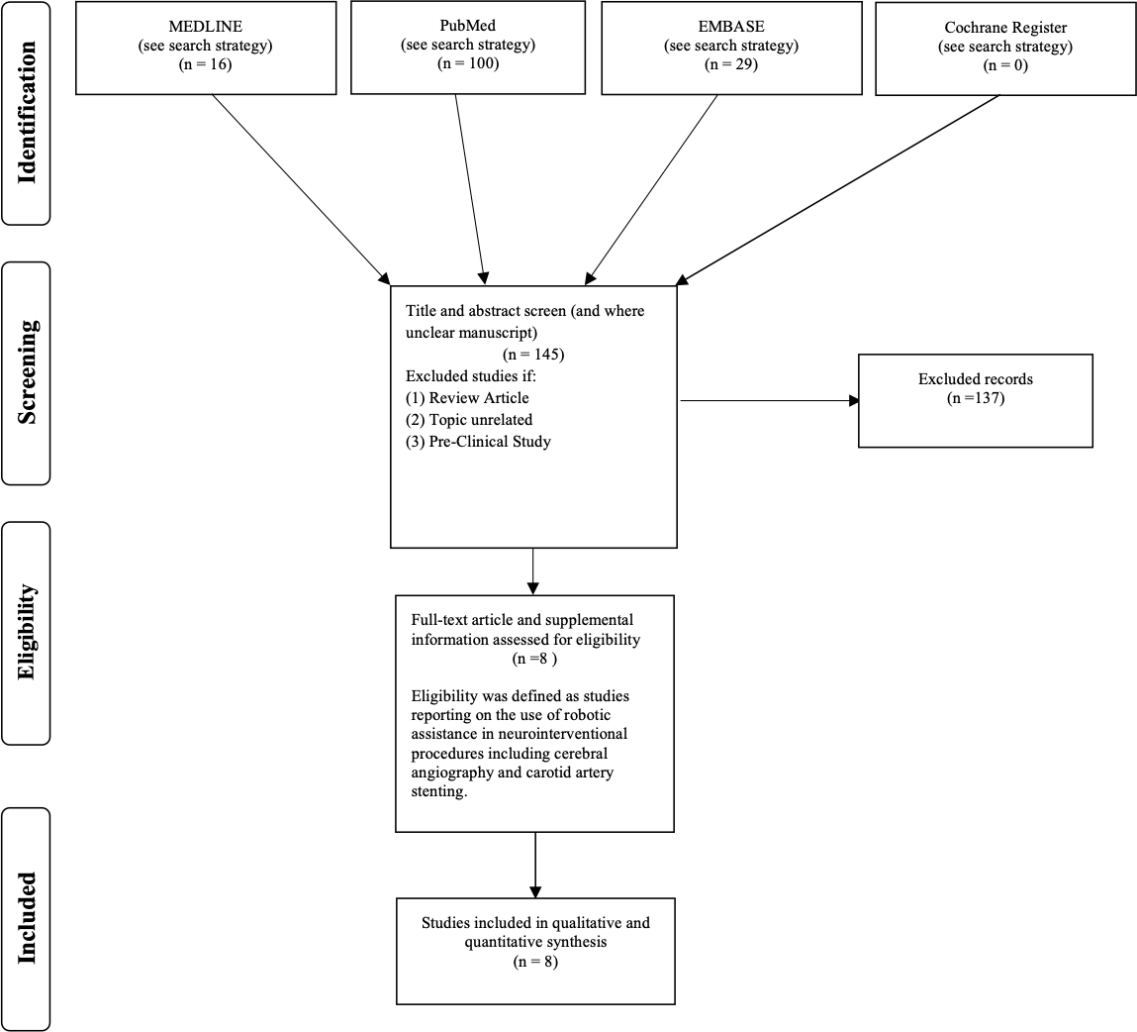
Preferred Reporting Items for Systematic Reviews and Meta-Analyses (PRISMA) flow diagram shows the number of articles searched and excluded at each stage of the literature search after screening titles, abstracts, and full texts.

Eligible articles were assessed for risk of bias and applicability using the QUADAS-2 methodology.^21^ We recorded which robotic system was used, the number of patients treated, and which procedure was performed. We recorded the technical success (successful robotic completion of the procedure as intended without manual takeover) and clinical success (successful completion of the procedure without complication leading to morbidity or mortality) for all procedures performed. We also recorded the stages of the procedure which were manually and robotically performed. The PRISMA flow diagram depicting the search results from each database can be seen in figure 4. One hundred and forty-five articles were identified across all database searches and the title and abstract were screened for inclusion. Eight articles were identified which fulfilled our eligibility criteria and were included in our qualitative and quantitative analysis. The articles are listed in table 2 which detail the number of patients treated, robotic system used, and the procedural stages that were manually and robotically performed.

**Table 2:** Articles included from the Preferred Reporting Items for Systematic Reviews and Meta-Analyses (PRSIMA) search strategy and information from the articles including the number of patients treated, robotic system used, and the procedural stages that were manually and robotically performed

Study	Robotic system	Procedures performed	Robotically performed stages	Manually performed stages
Lu *et al* 2016^22^	VIR-2 robot	15 DCA	Navigation of catheters and wires to target site from femoral sheath	Insertion of femoral sheath
Vuong *et al* 2017^23^	Magellan robotic system	9 DCA, 18 robot-assisted interventions (unspecified)	Navigation of catheters and wires to target site from femoral sheath	Insertion of femoral sheath
Jiang *et al* 2018^24^	Robot of endovascular treatment (RobEnt)	5 DCA	Navigation of catheters and wires to target site from manually placed catheter	Insertion of femoral sheath
Nogueira *et al* 2020^25^	Corpath GRX	4 CAS	Deployment and removal of distal embolic protection device Navigation of angioplasty balloon	Insertion of femoral sheath Selective catheterization of distal common carotid artery with guidewire and catheter Balloon angioplasty inflation Navigation and deployment of stent
Sajja *et al* 2020^18^	Corpath GRX	7 DCA, 3 CAS	Navigation of catheter to target vessel from aortic arch Deployment and removal of distal embolic protection device Navigation of angioplasty balloon and stent	Insertion of radial sheath Navigation of catheter and guidewire to descending aorta Balloon angioplasty inflation Stent deployment
Weinberg *et al* 2020^26^	Corpath GRX	6 CAS	Navigation of catheter to target vessel from aortic arch Deployment and removal of distal embolic protection device Navigation of angioplasty balloon and stent	Insertion of radial sheath Navigation of catheter and guidewire to descending aorta Balloon angioplasty inflation Stent deployment
Jones *et al* 2021^27^	Magellan robotic system	13 CAS	Navigation of catheters and wires to target site from femoral sheath	Insertion of femoral sheath Deployment of all procedural devices
Mendes Pereira *et al* 2020^28^	Corpath GRX	One stent-assisted aneurysm coiling	Navigation of microwire and catheter to right P1 from V4 Deployment of self expanding stent Navigation of microcatheter through stent into aneurysm sac Deployment of coils within the aneurysm cavity	Insertion of femoral sheath to right subclavian artery Selective catheterization of right V4 segment of vertebral artery.

CAS, carotid artery stenting; DCA, diagnostic cerebral angiography.

## Results

A total of eight eligible articles treating 81 patients were identified. Seven studies reported the use of robotic systems for diagnostic cerebral angiography or CAS^18 22–27^ and one case report described the use of a robotic system performing intracranial intervention.^28^ Two case series reported the use of experimental robotic systems,^22 24^ two case series used the Magellan system^23 27^ and the remaining studies used the Corpath GRX.^18 25 26 28^ In only three cases was manual conversion required, giving a technical success rate of 96% (78/81). There were no pertinent safety data (morbidity or mortality) or complications reported in any of the studies with a clinical success rate of 100%. Only one study performed a comparison between robotically assisted and completely manual procedures, which found a significant increase in the procedure time for robotic CAS.^26^

All identified evidence falls into the category of level 4 evidence,^23^ and thus currently, there is no high-level evidence to demonstrate that robotically performed neurointervention is at least non-inferior to manual procedures. QUADAS-2 methodology gave 100% (8/8) high or unclear ‘risk of bias’ and ‘concerns regarding applicability’ in all domains. No studies had clearly defined patient selection criteria, reference standards, or index tests. Despite the low level of evidence, there is value in discussing these individual studies as they represent the current state of the art and form a baseline for further research.

Lu *et al* 22 describe the testing of an experimental controller-responder vascular interventional robot (VIR-2) to perform cerebral angiography. Fifteen patients underwent successful cerebral angiography without complication. Details of the specific mechanics of the robot are not available. Jiang *et al* 24 similarly used a different experimental robotic system to perform five cerebral angiography procedures without complication.

The Magellan robotic system has been used in two studies to perform cerebral angiography and CAS, respectively. One group reported 27 patients undergoing cerebral angiography or providing some assistance in neurointerventional procedures.^23^ After attaching the robot to a short femoral sheath the robot was able to successfully navigate the catheter tip to the desired target for angiography or intervention. When robotic angiography was compared with historical manually performed procedures, there was no significant difference in procedure time, fluoroscopy time, or contrast volume and there were no complications. The Magellan system was subsequently used to perform CAS in 13 patients with technical and clinical success achieved in all cases.^27^ The robotic system was used to navigate a guide catheter to the carotid lesion and the intervention was performed manually.

The Corpath GRX has been used to perform seven diagnostic cerebral angiography procedures and 10 CAS procedures.^18 25 26^ These extracranial procedures were performed ‘on label’ as peripheral vascular interventions, but the authors noted some potential limitations to use of the devices for intracranial intervention. In three of the cerebral angiogram procedures, manual takeover was required due to presence of a bovine arch making navigation challenging. The interventionalists required the use of 0.035 and 0.038 inch glidewires (Terumo Medical Incorporation, Somerset, USA), which the robotic platform is unable to use.^18^ The authors in all studies manually performed balloon angioplasty and deployment of stents for the carotid interventions. The robot is able to navigate ‘over the wire’ equipment to the desired location for deployment; however, there is no mechanism for deploying devices robotically, which precludes the robotic use of many modern neurointerventional devices.^18^ One group compared robotic and manual CAS and found robotic CAS to have a significantly longer mean procedure duration, but there were no significant differences in fluoroscopy time, radiation exposure, or complications in their small sample of six robotically treated and seven manually treated patients.^26^

The Corpath GRX underwent preclinical testing to assess its feasibility to perform neurovascular intervention. The robot was successfully used in vitro in an aneurysm flow model (PAVM-44 Neuro Aneurysms, DialAct Corporation, USA) to navigate a microcatheter, deploy a bare-metal stent, and deliver two different coils.^29^ The research advanced to a porcine model: a 6 Fr sheath was manually placed into the common carotid artery and the Corpath GRX was used to robotically navigate a microcatheter into the porcine external carotid vasculature, a caliber similar to human intracranial vasculature, and coiling was performed within the rete mirabile successfully. This was the first study to demonstrate a robotic platform that can manipulate 2.4 Fr and 1.7 Fr microcatheters required for neurointervention. In a separate study the Corpath GRX was used in a porcine model to deliver Onyx (Medtronic, Irvine, USA) to embolize the rete mirabile. This was performed bilaterally in two swines with complete obliteration of the rete in all attempts, suggesting the platform could be feasible for arteriovenous malformation embolization.^30^

This preclinical testing with the Corpath GRX led to further modifications to improve its capability for neurointervention.^31^ A software modification termed ‘active device fixation’ was developed to allow independent movement of the microcatheter while keeping the guidewire or device fixed; the aim of this is to prevent unwanted movements of the guidewire tip or device, which could lead to perforation of neurovascular structures. They also made mechanical changes to the cassette based on observations from previous trials with the robot. In previous studies they noticed first, buckling of the smaller caliber microcatheters within the cassette and second, that certain devices such as coil systems were too short to be inserted into the guidewire track of the cassette. Therefore, respectively, they incorporated a cover over the connection of the robotic arm to the sheath to prevent buckling and they modified the guidewire track to be compatible with coiling systems. The authors reported successful navigation of the microcatheter to an induced aneurysm and to the rete mirabile without complication. The active device fixation reduced the amount of forward motion of the guidewire on microcatheter manipulation and there was no instance of buckling of the microcatheter from the cassette, suggesting that the modifications had achieved a safer platform for neurointervention. We have included video footage of the robotic system being used in an unpublished case to treat a basilar aneurysm (online supplemental video 1), which demonstrates the improved capability of the system for neurointervention while also showing how an operator is able to robotically manipulate a microcatheter, guidewire, and neurointerventional devices.

10.1136/neurintsurg-2021-018096.supp3 Supplementary video

10.1136/neurintsurg-2021-018096.supp4 Supplementary data

Following these engineering adaptations, a robotically performed, in human neurovascular intracranial procedure was performed using the updated Corpath GRX system.^28^ A patient with a sidewall distal basilar aneurysm was treated with a stent-assisted coiling procedure. The team spent 30 hours familiarizing themselves with the equipment and performed two full procedure rehearsals on patient-specific flow models based on 4D CT angiography models. A 6 Fr sheath was manually placed in the right subclavian artery followed by an intermediate catheter in the right V4 segment when the robotic cassette was attached. With the use of the robot, a 1.7 Fr microcatheter was advanced to the right P1 segment over a 0.014 inch microwire. From this position the microwire was replaced with a self-expanding stent, which was deployed across the aneurysm neck. The microcatheter was successfully placed within the aneurysm, and 14 coils were inserted successfully to occlude the aneurysm sac. The procedure occurred without complication; however, the cassette had to be replaced after deploying eight coils owing to a mechanical failure. Follow-up magnetic resonance angiography 2 weeks postprocedure showed complete aneurysm occlusion (Raymond scale 1).

### Limitations of current systems and future challenges

A current drawback with all commercially available robotic platforms is the lack of haptic feedback—that is, the lack of tactile feedback to the operator from the controller. Catheters and guidewires undergo three forces that are transmitted to the operator’s hands when performing interventional procedures manually: viscous forces between the catheters and the blood; friction forces between the catheters and the vessel wall; and impact forces from the tips of the catheter and guidewire with the vessel wall. Operators use tactile feedback from the combination of these forces to navigate safely through the intracranial vasculature and to safely deploy devices. Currently, no commercially available robotic platform provides haptic feedback to the operator.

For an effective haptic feedback system there are two requirements

- A mechanism to measure or estimate forces applied to the catheter and guidewire during the procedure.
- A mechanism in the controller system to allow the operator to appreciate these forces.

There have been numerous experimental efforts to measure and estimate these forces using sensors in the catheter tip,^32^ sensing mechanisms within the responder system,^33 34^ or model/image-based haptic feedback.^35^ Additionally, haptic feedback is frequently incorporated into commercially available endovascular simulators, and forces are estimated using mathematical models based on the interaction between catheters, guidewires, and blood vessels.^36^ While many of these techniques can produce accurate force estimations, the mechanism within the controller system to supply these forces to the operator is variable. Several researchers have used commercially available haptic controllers such as the six degrees of freedom phantom devices (3D Systems, South Carolina, USA),^37 38^ which comprise a stylus controller similar to the joystick controllers of commercial devices. Forces applied through this sort of controller system are not directly matched to the forces felt by interventionalists when manipulating catheters and guidewires. A controller system that allows the operator to control the robot with movements equivalent to manual procedures (advancing and rotating) would serve as an ideal platform on which an interventionalist could appreciate haptic feedback and relate it to their previous manual experience. Several researchers have designed such experimental controllers and found that this type of system was capable of producing accurate catheter movements to within 1 mm of linear motion and 1 degree of rotation.^39^ Furthermore, when incorporated with haptic feedback, these controllers can reduce the amount of force applied to the walls of an aortic vascular phantom in comparison with manual manipulation.^40^

Another drawback is the lack of sensory feedback when administering contrast, which is important to optimize angiographic images and prevent vascular injury as contrast administered at high pressure may lead to the rupture of intracranial aneurysms.^41^ Haptic syringe devices have recently been incorporated into experimental angiographic simulators,^42^ although this is yet to be a feature in robotic systems. While a robotic platform that reliably performs neurointervention is likely to require haptic feedback, further preclinical studies are needed to determine the optimum method for producing haptic feedback as well as operator validation studies which demonstrate improved procedural performance with haptic feedback.

An additional concern is that the learning curve for robotically performed neurointervention is unknown with only anecdotal evidence (level 4)^23^ from the studies in this review. Only two studies reported the training required before the procedure, which involved the use of commercial simulators and procedure rehearsal on patient-specific phantoms.^25 28^ Further research is required to understand the learning curve for robotically performed surgery across all surgical subspecialties, with a recent systematic review unable to identify any high-level evidence for appropriate training before robotically performed procedures.^43^ Several studies have shown that training using endovascular simulators can improve procedural performance, including reducing procedure time, fluoroscopy time, and contrast volume,^44^ and therefore it is likely that standardized simulation-based training will be important for operators training in robotic neurointervention. Further investigation is needed to identify the appropriate training required before operators can be deemed to perform robotic procedures safely.

A key limitation of all current robotic platforms is the requirement for manual assistance at different stages of the procedure. The VIR-2 system, RobEnt system, and Magellan system are capable of navigation of a guide catheter from the femoral artery to the carotid artery for angiography,^22–24^ but the Corpath GRX system requires manual placement of the catheter close to the target site for intervention.^18 25 26 28^ Furthermore, current iterations of the Corpath GRX do not allow the robotic deployment of devices^18^ or the robotic inflation of balloons for angioplasty.^18 25^ A robot capable of performing remote robotic MT, for example, would require navigation of catheters and guidewires from a femoral or radial sheath, deployment of stent retriever devices, and the ability to perform stenting or angioplasty procedures if required. Related to this there are several key issues that require further investigation. For operators who perform manipulations to equipment, such as manually placing curves on microwires or steam-shaping microcatheters, there may be unforeseen problems with true remote intervention that may require additional specialized devices or training for technicians present in the procedural room. In one study, manual takeover was required for the navigation of a catheter in a bovine aortic arch,^18^ and there was one report of a robotic cassette failure requiring replacement.^28^ This raises concerns for the safety of teleoperated robotic neurointervention, where manual takeover will not be readily available. As previously mentioned, the technical success in the PRECISE trial was 98.9%,^45^ and in large-scale studies with laparoscopic surgical robotic systems, the device failure rate is reported as 0.38%.^46^ Further engineering modifications, followed by clinical trials, are needed to demonstrate similar reliability and safety of robotic systems for remote neurointervention without expert neurointerventional manual assistance. Promisingly there have been recent studies using the Corpath GRX system to successfully perform long-distance teleoperated PCI,^47^ and recent successful reports of the first teleoperated laparoscopic procedure using a 5G internet connection.^48^ While these procedures were performed with trained operators on site to perform certain manual stages and take over if required, the achievements, particularly for telecommunications, support the potential for teleoperated neurointervention if an efficacious robotic platform has been developed and undergone rigorous in-human investigation.

A major challenge to the uptake of robotic systems in neurointerventional surgery is the expected cost and training of staff. A robotic system for neurointervention is likely to be expensive for healthcare institutions, with the current Corpath GRX system costing in the region of US$600 000.^18^ There are also potential additional costs—for example, disposable cassettes seen with Corpath robotic systems which cost US$300 per procedure,^18^ and there will be additional costs for proprietary catheters like those required for the Magellan robotic system. For comparison, the use of the da Vinci robot is significantly more expensive than manual alternatives and does not necessarily provide superior clinical outcomes.^49^ While, for example, modeling studies in the UK suggest that performing manual MT up to 24 hours after symptom onset is potentially cost effective,^50^ there is no health economic evidence at present that teleoperated robotic MT would be cost effective.

## Conclusion

It is plausible that robotically performed neurointerventional procedures will eventually benefit patients and reduce occupational hazards for staff; however, there is no high-level efficacy and safety evidence to support this assertion. If robust efficacy and safety evidence emerges, and if proved to be cost effective, one potential use would be for a fully functioning platform to perform teleoperated intervention which, if applied to MT in stroke, would accelerate the treatment of eligible patients in locations which are at a considerable distance from the operator.

Potential platforms currently require considerable refinement. First, to ensure that minimal interventional input is required by an operator within the operating room. Second, to allow the precise use of a wide range of neurovascular microcatheters and devices. Third, to develop haptic feedback systems that directly match manual operator movements; this has the potential to reduce training time, make use of the operator’s pre-existing skills, and mitigate risks from catheter and wire damage through the appreciation of subtle catheter and wire movements.

10.1136/neurintsurg-2021-018096.supp5 Supplementary data
