# Supplementary material for: Robotics in neurointerventional surgery: a systematic review of the literature
Source: J Neurointerv Surg. 2021 Nov 19;14(6):539–45. doi: 10.1136/neurintsurg-2021-018096 (PMC9120401; doi:10.1136/neurintsurg-2021-018096)
Supplement: Supplementary data [file neurintsurg-2021-018096supp001.pdf]

Term	Definition
Controller-Responder	A model of communication in robotic systems whereby the 'Responder' unit copies the input to the 'Controller' unit exactly
Steerable Catheter/Sheath	A catheter that is manipulated by a mechanism to allow controlled deflection of the distal portion of the catheter or sheath
Degrees of Freedom	The number of possible independent directions of movement
Tendon Drive	Use of straps embedded along the length of the catheter to allow deflection of catheter tip under robotic control
Linear Motion	Advancement or retraction of catheter or guidewire
Single Use Sterile Cassette	Plastic sterile container mounted on robotic arm where driving components for the catheters and guidewires are contained
Haptic Feedback	Tactile feedback from the controller system to the operator
Caterpillar-like control system	Robotic system for controlling catheter tendons to allow tip deflection

Supplementary Table 1: Definitions of robotic terminology for the clinician used within this review.
