# Supplementary material for: Robotics in neurointerventional surgery: a systematic review of the literature
Source: J Neurointerv Surg. 2021 Nov 19;14(6):539–45. doi: 10.1136/neurintsurg-2021-018096 (PMC9120401; doi:10.1136/neurintsurg-2021-018096)
Supplement: Supplementary data [file neurintsurg-2021-018096supp002.pdf]

Supplementary Appendix S1. PubMed, MEDLINE, EMBASE and Cochrane Register search strategies. Recommendations for a sensitive search with low precision; with subject headings with exploded terms; and with no language restrictions, were followed [1].

MEDLINE (OVID). PubMed was included.

The search strategy for Title/Abstract terms used a combination of subject headings (MeSH terms) and keywords:

Database: Ovid MEDLINE(R) ALL <1946 to April 12 2021>

Search Strategy:

----

1 exp Robotics (30265)

2 interventional neuroradiology (669)

3 neurointervention (259)

4 mechanical thrombectomy (3685)

5 carotid artery stenting (2068)

6 exp cerebral angiography (34008)

7 2 or 3 or 4 or 5 or 6 (41032)

8 1 and 7 (16)

EMBASE (OVID).

Subject headings and keywords:

Database: Embase <1974 to 2021 April 12>

Search Strategy:

1 exp Robotics (42604)

2 interventional neuroradiology (1280)

3 neurointervention (450)

4 exp mechanical thrombectomy (8445)

5 exp carotid artery stent (1224)

6 exp brain angiography (26955)

7 2 or 3 or 4 or 5 or 6 (37544)

8 1 and 7 (29)

Cochrane Register.

Subject headings and keywords:

Date Run: 22/02/2020 14:00

ID	Search	Hits
#1	MeSH descriptor: [Robotics] explode all trees	873
#2	neuroradiology	981
#3	mechanical thrombectomy	532
#4	carotid artery stent	530
#5	cerebral angiography	1184
#6	{OR #2-#5}	2903
#7	#1 AND #6	0

PubMed (HDAS).

Subject headings and keywords:

Database: PubMed <All performed 16/04/2021>

Search Strategy:

1 robot or robotic (62073)

2 interventional neuroradiology (5575)

3 neurointervention (957)

4 mechanical thrombectomy (3675)

5 carotid artery stenting (3068)

6	cerebral angiography (51588)
7	2 or 3 or 4 or 5 or 6 (62321)
8	1 and 7 (100)
