# Supplementary material for: Robotics in neurointerventional surgery: a systematic review of the literature
Source: J Neurointerv Surg. 2021 Nov 19;14(6):539–45. doi: 10.1136/neurintsurg-2021-018096 (PMC9120401; doi:10.1136/neurintsurg-2021-018096)
Supplement: Supplementary data [file neurintsurg-2021-018096supp004.pdf]

Supplementary Video Legend:

Accelerated video demonstration of the use of the Corpath GRX System (Corindus, Waltham MA) to perform stent-assisted coiling of a basilar aneurysm.
00:00 – 01:05 – The robotic system is used to navigate a microcatheter (Excelsior SL-10) and guidewire (Synchro-14) past the aneurysm neck.
01:05 – 01:50 – The robotic cassette is opened and the guidewire is removed. A self-expanding stent (Neuroform Atlas 4.5 mm x 21 mm) is placed into the device track.
01:50 – 03:30 – The stent is deployed across the aneurysm neck. The operator can be seen to use the joysticks as well as touch screen controls which allow individual 1mm movements of the device or catheter.
03:30 – 05:00 – The robotic system is used to navigate a microcatheter into the aneurysmal sac. The device track is then used to robotically deploy 4 coils (Axium Prime) for successful occlusion of the aneurysm.

Procedure performed by Dr Vitor Mendes Pereira.
